# Allometric scaling of dietary linoleic acid on changes in tissue arachidonic acid using human equivalent diets in mice

**DOI:** 10.1186/1743-7075-8-43

**Published:** 2011-06-24

**Authors:** Kylie A Weldon, Jay Whelan

**Affiliations:** 1 Department of Nutrition, 1215 West Cumberland Avenue, 229 Jessie Harris Building, University of Tennessee, Knoxville, TN 37996-1920, USA

## Abstract

**Background:**

It is hypothesized that dietary linoleic acid (LA) promotes chronic and acute diseases in humans by enriching tissues with arachidonic acid (AA), its downstream metabolite, and dietary studies with rodents have been useful for validation. However, levels of LA in research diets of rodents, as published in the literature, are notoriously erratic making interspecies comparisons unreliable. Therefore, the ability to extrapolate the biological effects of dietary LA from experimental rodents to humans necessitates an allometric scaling model that is rooted within a human equivalent context.

**Methods:**

To determine the physiological response of dietary LA on tissue AA, a mathematical model for extrapolating nutrients based on energy was used, as opposed to differences in body weight. C57BL/6J mice were divided into 9 groups fed a background diet equivalent to that of the US diet (% energy) with supplemental doses of LA or AA. Changes in the phospholipid fatty acid compositions were monitored in plasma and erythrocytes and compared to data from humans supplemented with equivalent doses of LA or AA.

**Results:**

Increasing dietary LA had little effect on tissue AA, while supplementing diets with AA significantly increased tissue AA levels, importantly recapitulating results from human trials.

**Conclusions:**

Thus, interspecies comparisons for dietary LA between rodents and humans can be achieved when rodents are provided human equivalent doses based on differences in metabolic activity as defined by energy consumption.

## Background

As surrogates for human inquisition, animal models reside at the core of medical innovation. Through careful environmental control, these genetically similar models facilitate therapeutic advancements in the magnitude of human disease. Rodent dietary composition is of particular interest in the field of nutrition research as it provides a way to assess the translational ability of individual dietary constituents, through appropriate dosing of nutrients, to physiological effects observed in humans consuming similar levels of nutrients.

Dietary profiles of n-6 polyunsaturated fatty acid (PUFA), linoleic acid (LA) and the relationship to chronic and acute diseases, in both rodents and humans, appears to lie in tissue enrichment of the downstream metabolite, arachidonic acid (AA) [1-3]. It is hypothesized that metabolism of dietary AA produces bioactive compounds called eicosanoids that are positively correlated with the appreciation of tissue AA [4]. While the relationship of AA and eicosanoids is well established, the response to dietary LA on changes in tissue levels of AA, within the context of a human equivalent diet, remains inconclusive.

The inconsistent use of n-3 and n-6 essential fatty acids (EFAs) in the background of rodent diets is pervasive in the literature [5-10]. These EFAs are important components of the Western diet and can impact the AA phospholipid pool when absent or provided at insufficient quantities in the diet. Despite suggestions otherwise, a systematic review of the human literature reports that increases in dietary LA do not appear to significantly modify AA levels in phospholipids of plasma/serum or erythrocytes when supplemented to standard Western diets [11]. Therefore, if precise physiological nutrient translation of fatty acids is desired, it may be important for dietary aspects of the rodent model to bear firm resemblance to human dietary components.

This study was designed to investigate a putative standard for allometric scaling with regards to an animal dietary design as it relates to the relationship between dietary LA and tissue AA. This is the first study to examine the physiological response of dietary LA on changes in AA levels in plasma/serum or erythrocyte phospholipids when provided at human equivalent supplemental doses within the context of a Western background diet, based on a percentage of energy (i.e., metabolic activity). We further investigated the potential contribution of dietary AA on tissue AA content within the context of a Western-type diet. This mathematical model using a surrogate of metabolic activity instead of differences in BW for allometric scaling should better equate interspecies translation and accommodate the differences in metabolic disparity between rodents and humans.

## Methods

### Animals

Sixty-two *C57BL/6J *male mice (Harlan Laboratory, Indianapolis, IN), 6-7 weeks of age, were randomly assigned to nine dietary groups; 5-7 animals per group were housed 2-3 animals per cage in a temperature controlled room with a 12 hr light-dark cycle. Prior to sacrifice, animals were fasted overnight. All animal procedures were approved by the University of Tennessee Animal Care and Use Committee in accordance with NIH guidelines.

### Diets

All animals were maintained on a control diet for one week prior to being transferred to one of the experimental diets or maintained on the control diet. The control diet was based on a US17 Monsanto diet with slight modifications in macronutrient distributions (Table 1). The diet was designed to mimic the Western diet with the following distribution (% of energy): protein 16%, carbohydrates 50% and lipids 34% (Research Diets, New Brunswick, NJ) [12]. Within the lipid fraction, saturated, monounsaturated and polyunsaturated fats were designed to be provided at 13%, 14% and 7% of energy, respectively. The polyunsaturated fats LA, ALA, AA and EPA+DHA were provided at 6%, 0.6%, 0.07% and 0.1% of energy, respectively. These levels are similar to those suggested in the literature for humans on a Western diet [12,13] and/or supported by the DRIs for median daily intakes [14]. AA and EPA+DHA were provided as ethyl esters (NuChek Prep, Elysian, MN). Experimental diets remained isocaloric and were formulated using the control diet as the background diet containing LA at ~6% of energy with additional adjustments in LA content (-2%, +2%, +4%, +6%, +8% of energy) with the addition (or subtraction) of sunflower oil (70% w/w LA) at the expense of cocoa butter, palm and trisun oils based on the ratios found in the control diet. The diets supplemented with AA were adjusted at the expense of cocoa butter.

**Table 1:** Composition of the diets

	Dietary Groups								
	
	1	2	3	4	5	6	7	8	9
Diet	-2%^1 ^LA	Control	+2% LA	+4% LA	+6% LA	+8% LA	+0.23% AA	+0.45% AA	+1.36% AA
	g/100 g								
	
Protein	17.4	17.4	17.4	17.4	17.4	17.4	17.4	17.4	17.4
Carbohydrate	54.7	54.7	54.7	54.7	54.7	54.7	54.7	54.7	54.7
Lipid	16.8	16.8	16.8	16.8	16.8	16.8	16.8	16.8	16.8

	g/kg								
	
Casein	171	171	171	171	171	171	171	171	171
L-Cysteine	3	3	3	3	3	3	3	3	3
Corn Starch	337	337	337	337	337	337	337	337	337
Maltodextrin 10	85	85	85	85	85	85	85	85	85
Sucrose	114	114	114	114	114	114	114	114	114
Cellulose	57	57	57	57	57	57	57	57	57
Cocoa Butter, Deodorized	47.5	42.57	38.12	33.4	28.72	23.99	41.47	40.37	35.97
Flaxseed Oil	5.11	5.11	5.11	5.11	5.11	5.11	5.11	5.11	5.11
Palm Oil, Bleached, Deodorized	66.5	59.6	53.38	46.8	40.23	33.62	59.6	59.6	59.6
Safflower Oil, USP	17.75	32.35	32.35	32.35	32.35	32.35	32.35	32.35	32.35
Trisun Extra	34.2	30.65	27.46	24.06	20.69	17.29	30.65	30.65	30.65
Sunflower Oil	-	-	14.6	29.30	43.90	58.60	-	-	-
Arachidonic Acid, Ethyl Ester	0.40	0.40	0.40	0.40	0.40	0.40	1.10	2.2	6.6
Eicosapentaenoic Acid, Ethyl Ester	0.17	0.17	0.17	0.17	0.17	0.17	0.17	0.17	0.17
Docosahexaenoic Acid, Ethyl Ester	0.27	0.27	0.27	0.27	0.27	0.27	0.27	0.27	0.27

^1^% change with regards to energy. In addition, the following were also included in the diet but are not listed above (g/1000 g): mineral mix S10026,11 g; dicalcium phosphate, 15 g; calcium carbonate 6.2 g; potassium citrate, 1 H_2_O, 18.7 g; vitamin mix V13401, 11.4 g; alpha-tocopheryl acetate (500 IU/g), 0.1 g.

Abbreviations: AA, arachidonic acid; LA, linoleic acid

Water and food were provided ad libitum for 21-25 days. Fresh diets were provided daily and uneaten food was discarded to minimize oxidation prior to consumption. Fatty acid analysis of the diets is presented in (Table 2).

**Table 2:** The fatty acid composition of the diets

	Dietary Groups								
	
	1	2	3	4	5	6	7	8	9
	-2%^1^		2%	4%	6%	8%	0.23%	0.45%	1.36%
Fatty Acids	LA	Control	LA	LA	LA	LA	AA	AA	AA
12:0	0.47^2^	0.42	0.39	0.34	0.27	0.26	0.42	0.43	0.42
14:0	0.69	0.69	0.59	0.56	0.48	0.43	0.63	0.65	0.66
16:0	26.48	24.69	22.66	20.63	18.71	16.8	24.62	24.31	23.71
16:1	0.17	0.1	0.15	0.14	0.09	0.09	0.15	0.16	0.16
18:0	13.13	11.39	11.19	10.08	9.52	8.5	11.69	11.29	10.54
18:1n-9	42.44	40.44	38.44	36.62	35.00	33.06	40.11	39.79	39.00
18:2n-6	13.24	18.93	23.23	28.24	32.5	37.39	19.01	19.05	19.08
18:3n-3	1.78	1.79	1.77	1.8	1.77	1.8	1.78	1.84	1.83
20:0	0.58	0.54	0.52	0.49	0.48	0.44	0.55	0.55	0.51
20:1	0.14	0.17	0.15	0.15	0.17	0.14	0.16	0.17	0.14
20:4n-6	0.21	0.21	0.22	0.22	0.2	0.22	0.55	1.11	3.32
20:5n-3	0.09	0.03	0.08	0.07	0.06	0.1	0.05	0.09	0.06
22:0	0.31	0.31	0.34	0.37	0.42	0.46	0.31	0.29	0.30
22:6n-3	0.26	0.29	0.27	0.28	0.29	0.31	0.3	0.27	0.27

^1^% change with regards to energy

^2^g/kg diet

Abbreviations: AA, arachidonic acid; LA, linoleic acid.

### Fatty acid analysis

Animals were randomized on a 5 day sacrifice cycle. Following 21-25 days on the experimental and control diets, 0.5-1.0 ml of whole blood was collected via cardiac puncture under anesthesia (isofluorane inhalation) using a tuberculin syringe with a 25 gauge needle containing an anticoagulant (3.8% trisodium citrate). Whole blood was centrifuged at 660 × g for 4 min at room temperature for separation of plasma and a pellet that was predominately erythrocytes, where each fraction was subjected to lipid extraction. Three ml of chloroform-methanol (1:2, v/v) were added to each fraction, and lipids were extracted with chloroform (1 ml) plus saline (1 ml), followed by chloroform (1 ml) (2×). The pooled chloroform extracts were evaporated and resuspended in a small amount of chloroform (~25 μL), and phospholipids were separated via thin layer chromatography (TLC) using HPTLC plates precoated with silica gel 60 (Merck, Darmstadt, Germany) using a chloroform-methanol (8:1, v/v) solvent system. The phospholipids were recovered from the TLC plates and saponified in 0.5 N NaCl and in the presence of BF_3 _in methanol at 86°C. Fatty acid methyl esters were extracted with equal volumes of hexane (2×) and evaporated under nitrogen. Fatty acid methyl esters were resuspended in hexane and analyzed by gas chromatography with a Hewlett-Packard 5880 gas chromatograph (Rochester, NY) using a DB23 capillary column (0.25 mm × 30 m) (J and W Chromatography, Folsom, OH) with hydrogen as the carrier gas, with temperature programming from 160°C to 250°C at 3.5°C/min The internal standard 1,2 diheptadecanoyl-sn-glycero-3-phosphocholine (17:0) (Avanti Polar Lipids, Alabaster, AL) was added to each sample prior to lipid extraction. The fatty acid methyl esters were identified by comparing the retention times with those of known standards (NuChek Prep, Elysian, MN). The fatty acids are presented as mole %.

### Statistical analysis

Phospholipid fatty acid content in plasma and erythrocytes were compared across treatment groups using a one-way analysis of variance (ANOVA), followed by Tukey's Honestly Significant Difference (HSD) post-hoc test to determine significant differences between groups. All data were tested for normality, homogeneity of variance, and for outliers. The data were evaluated by SPSS 18 statistical package (University of Tennessee, Knoxville, TN). Data was considered significant at p < 0.05.

## Results

Food intake and weight gain were not statistically different between dietary groups (data not shown).

### Fatty acid composition of plasma phospholipids

**Figure 1:**
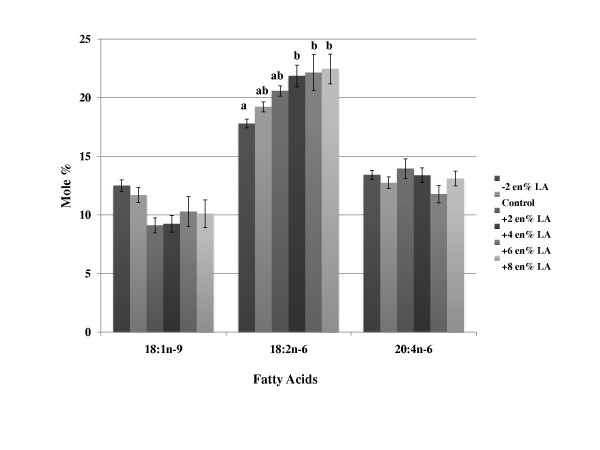
**Effects of increasing/decreasing dietary linoleic acid on changes in plasma/serum phospholipid fatty acid concentration**. Mice were fed background diets that mimicked the composition of a Western diet with increasing or decreasing levels (% change, based on energy) of linoleic acid. The data (mole %) is presented as mean ± SD. Means with the same superscript within the same row (i.e., individual fatty acid) are not statistically different at (p < 0.05). Groups of bars within each fatty acid without superscripts indicate no significant differences were observed among groups. Abbreviations: LA, linoleic acid.

**Figure 2:**
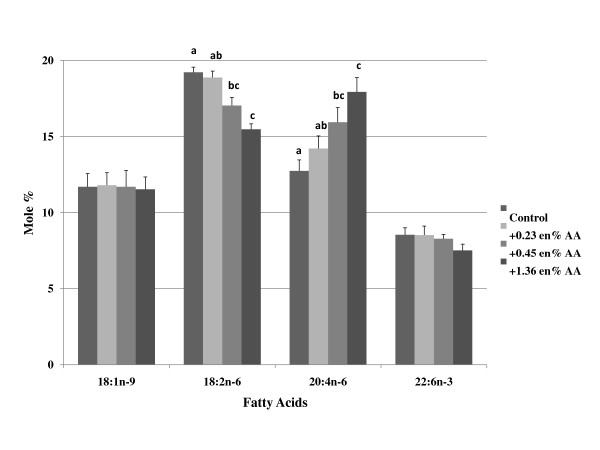
**Effects of increasing dietary arachidonic acid on changes in plasma/serum phospholipid fatty acid concentration**. Mice were fed background diets that mimicked the composition of a Western diet with increasing levels (% change, based on energy) of arachidonic acid. The data (mole %) is presented as mean ± SD. Means with the same superscript within the same row (i.e., individual fatty acid) are not statistically different at (p < 0.05). Groups of bars within each fatty acid without superscripts indicate no significant differences were observed among groups. Abbreviations: AA, arachidonic acid.

The composition of oleic acid and LA in plasma phospholipids tended to reflect differences in dietary levels of these fatty acids; however, much of these effects were not statistically significant (Figure 1 and Table 3). The dietary group with the lowest levels of LA and highest levels of oleic acid (group 1) had the lowest levels of LA and highest levels of oleic acid in the plasma phospholipids, respectively. The levels of AA did not change in any of the groups with increasing or decreasing levels of dietary LA (Table 3). DHA levels were not different among groups, with the exception of group 5. When AA was supplemented to the diets, tissue AA levels progressively increased in a dose responsive manner at the expense of LA (Figure 2 and Table 4), but tissue DHA levels did not change. A summary of the effects of LA and AA supplementation are provided in (Figure 1 and Figure 2), respectively.

**Table 3:** The fatty acid composition of plasma phospholipids from mice fed linoleic acid supplemented diets

	Dietary Groups					
	
Fatty Acid	1	2	3	4	5	6
	**-2% LA**^**1**^	Control	+2% LA	+4% LA	+6% LA	+8% LA
16:0	33.54 ± 0.93^2^	34.40 ± 0.80	33.60 ± 1.03	33.25 ± 0.96	35.29 ± 1.15	32.87 ± 0.88
18:0	13.68 ± 0.55	13.41 ± 0.31	13.07 ± 0.56	13.84 ± 0.61	13.97 ± 0.66	13.49 ± 0.54
18:1n-9	12.50 ± 0.50	11.69 ± 0.65	9.11 ± 0.64	9.23 ± 0.70	10.29 ± 1.26	10.10 ± 1.18
18:2n-6	17.80 ± 0.28^a^	19.22 ± 0.42^ab^	20.58 ± 0.44^ab^	21.86 ± 0.93^b^	22.15 ± 1.53^b^	22.46 ± 1.27^b^
20:4n-6	13.41 ± 0.40	12.74 ± 0.50	13.94 ± 0.84	13.39 ± 0.63	11.77 ± 0.75	13.10 ± 0.63
22:6n-3	9.07 ± 0.98^a^	8.54 ± 0.56^ab^	9.09 ± 0.60^a^	8.43 ± 0.59^ab^	6.53 ± 0.79^b^	7.99 ± 0.49^ab^

^1^%change with regards to energy

^2^Relative abundance (mol%) presented as mean ± SEM

^ab^Means with the same superscript within the same row are not statistically different at p < 0.05, Tukey's honestly significant difference.

Abbreviations: LA, linoleic acid

**Table 4:** The fatty acid composition of plasma phospholipids from mice fed arachidonic acid supplemented diets

	Dietary Groups			
	
	2	7	8	9
Fatty Acid	Control	**+0.23% AA**^**1**^	+0.45% AA	+1.36% AA
16:0	34.40 ± 0.87^2^	33.62 ± 0.82	33.71 ± 1.07	34.76 ± 0.81
18:0	13.41 ± 0.34	13.25 ± 0.42	13.34 ± 0.53	12.82 ± 0.36
18:1n-9	11.69 ± 0.72	11.80 ± 0.83	11.69 ± 0.96	11.53 ± 0.94
18:2n-6	19.22 ± 0.46^a^	18.88 ± 0.59^ab^	17.04 ± 0.28^bc^	15.47 ± 0.41^c^
20:4n-6	12.74 ± 0.54^a^	14.21 ± 0.66^ab^	15.94 ± 0.81^bc^	17.93 ± 1.00^c^
22:6n-3	8.54 ± 0.55	8.52 ± 0.52	8.28 ± 0.51	7.51 ± 0.45

^1^%change with regards to energy

^2^Relative abundance (mol%) presented as mean ± SEM

^abc^Means with the same superscript within the same row are not statistically different at p < 0.05, Tukey's honestly significant difference.

Abbreviations: AA, arachidonic acid

### Fatty acid composition of erythrocyte phospholipids

**Figure 3:**
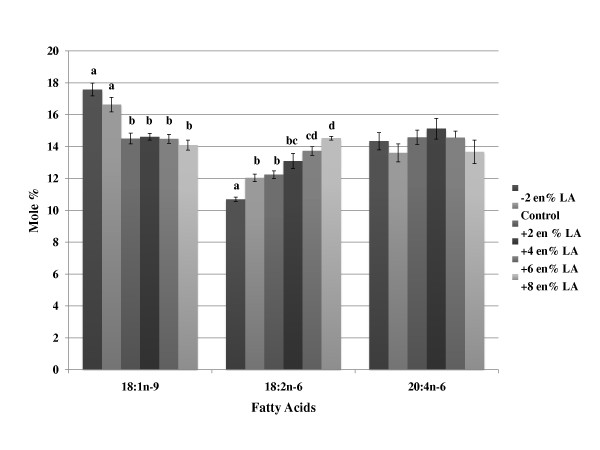
**Effects of increasing/decreasing dietary linoleic acid on changes in erythrocyte phospholipid fatty acid concentration**. Mice were fed background diets that mimicked the composition of a Western diet with increasing or decreasing levels (% change, based on energy) of linoleic acid. The data (mole %) is presented as mean ± SD. Means with the same superscript within the same row (i.e., individual fatty acid) are not statistically different at (p < 0.05). Groups of bars within each fatty acid without superscripts indicate no significant differences were observed among groups. Abbreviations: LA, linoleic acid.

**Figure 4:**
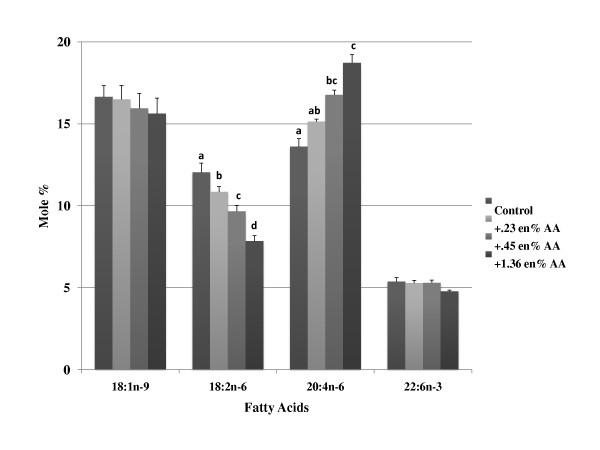
**Effects of increasing dietary arachidonic acid on changes in erythrocyte phospholipid fatty acid concentration**. Mice were fed background diets that mimicked the composition of a Western diet with increasing levels (% change, based on energy) of arachidonic acid. The data (mole %) is presented as mean ± SD. Means with the same superscript within the same row (i.e., individual fatty acids) are not statistically different at (p < 0.05). Groups of bars within each fatty acid without superscripts indicate no significant differences were observed among groups. Abbreviations: AA, arachidonic acid.

The composition of oleic acid and LA in the phospholipids of erythrocytes reflected differences in dietary levels of these fatty acids where LA supplementation significantly increased LA in the tissues (Table 5 and Figure 3). Levels of dihomo-gamma-linolenic acid (20:3n-6) and AA were unaffected by changes in dietary LA. Similarly, DHA content in erythrocytes were unaffected by changes in LA intake. When AA was supplemented in the diets, erythrocyte AA content, as well as that of its metabolites 22:4 n-6 and 22:5 n-6 progressively increased primarily at the expense of LA, but reductions in dihomo-gamma-linolenic acid were also observed (Table 6 and Figure 4). DHA levels were not reduced with increasing levels of dietary AA.

**Table 5:** The fatty acid composition of erythrocytes phospholipids from diets supplemented with linoleic acid

	Dietary Groups					
	
Fatty Acid	1	2	3	4	5	6
	**-2% LA**^**1**^	Control	+2% LA	+4% LA	+6% LA	+8% LA
16:0	34.19 ± 0.66^2^	34.80 ± 0.63	34.40 ± 0.48	32.81 ± 0.69	34.67 ± 0.62	33.57 ± 0.71
18:0	13.77 ± 0.30	14.09 ± 0.52	15.45 ± 0.43	14.73 ± 0.45	14.18 ± 0.30	15.35 ± 0.57
18:1n-9	17.57 ± 0.40^a^	16.63 ± 0.46^a^	14.50 ± 0.34^b^	14.61 ± 0.21^b^	14.48 ± 0.28^b^	14.09 ± 0.31^b^
18:2n-6	10.69 ± 0.13^a^	12.03 ± 0.23^b^	12.23 ± 0.24^b^	13.09 ± 0.48^bc^	13.72 ± 0.28^cd^	14.52 ± 0.12^d^
20:3n-6	1.18 ± 0.33	1.12 ± 0.04	1.22 ± 0.61	1.19 ± 0.08	1.18 ± 0.03	1.18 ± 0.06
20:4n-6	14.33 ± 0.54	13.60 ± 0.57	14.58 ± 0.45	15.12 ± 0.65	14.56 ± 0.41	13.66 ± 0.74
22:4n-6	1.40 ± 0.05	1.27 ± 0.13	1.44 ± 0.10	1.62 ± 0.13	1.67 ± 0.06	1.51 ± 0.10
22:5n-6	0.40 ± 0.05	0.45 ± 0.02	0.50 ± 0.03	0.58 ± 0.10	0.55 ± 0.02	0.47 ± 0.04
22:5n-3	0.70 ± 0.04	0.64 ± 0.04	0.60 ± 0.03	0.63 ± 0.05	0.60 ± 0.03	0.57 ± 0.05
22:6n-3	5.76 ± 0.34	5.37 ± 0.49	5.43 ± 0.20	5.64 ± 0.36	5.29 ± 0.27	4.99 ± 0.54

^1^%change with regards to energy

^2^Relative abundance (mol%) presented as mean ± SEM

^abcd^Means with the same superscript within the same row are not statistically different at p < 0.05, Tukey's honestly significant difference.

Abbreviations: LA, linoleic acid

**Table 6:** The fatty acid composition of erythrocyte phospholipids from diets supplemented with arachidonic acid

	Dietary Groups			
	
Fatty Acid	2	7	8	9
	Control	**+0.23% AA**^**1**^	+0.45% AA	+1.36% AA
16:0	34.80 ± 0.69^2^	34.08 ± 0.86	34.43 ± 0.91	35.17 ± 0.95
18:0	14.09 ± 0.57	14.56 ± 0.31	14.03 ± 0.37	13.95 ± 0.34
18:1n-9	16.64 ± 0.50	16.49 ± 0.15	15.94 ± 0.29	15.62 ± 0.51
18:2n-6	12.03 ± 0.25^a^	10.85 ± 0.16^b^	9.65 ± 0.18^c^	7.84 ± 0.07^d^
20:3n-6	1.12 ± 0.05^a^	0.94 ± 0.01^b^	0.77 ± 0.02^c^	0.44 ± 0.01^d^
20:4n-6	13.60 ± 0.63^a^	15.13 ± 0.52^ab^	16.77 ± 0.53^bc^	18.71 ± 0.93^c^
22:4n-6	1.27 ± 0.15^a^	1.54 ± 0.09^ab^	1.90 ± 0.07^bc^	2.20 ± 0.11^c^
22:5n-6	0.45 ± 0.03^a^	0.49 ± 0.02^ab^	0.57 ± 0.04^ab^	0.73 ± 0.05^c^
22:5n-3	0.64 ± 0.06	0.64 ± 0.03	0.65 ± 0.03	0.55 ± 0.04
22:6n-3	5.37 ± 0.54	5.28 ± 0.23	5.29 ± 0.37	4.78 ± 0.40

^1^%change with regards to energy

^2^Relative abundance (mol%) presented as mean ± SEM

^abcd^Means with the same superscript within the same row are not statistically different at p < 0.05, Tukey's honestly significant difference.

Abbreviations: AA, arachidonic acid

### Comparison of LA and AA data in the mouse to similar data generated in human clinical trials

**Figure 5:**
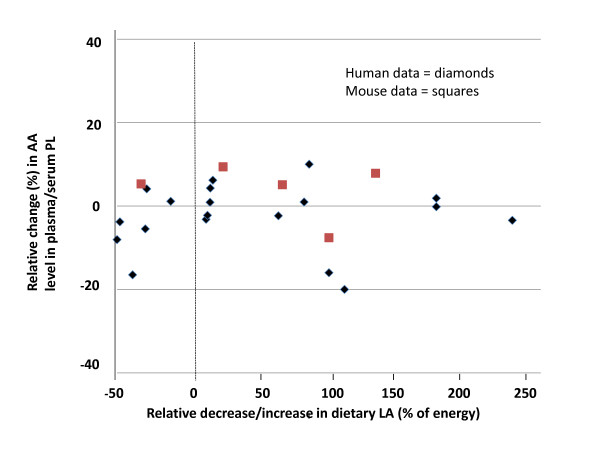
**Comparison of LA data in the mouse to similar data generated in human clinical trials; increasing levels of supplemented LA (% change based on energy) on changes in plasma/serum AA content**. The changes in AA levels of plasma phospholipids from this study (red squares) were plotted against archival data from human clinical trials (blue diamonds) (used with permission, see ref. 11). Abbreviations: AA, arachidonic acid; LA, linoleic acid; PL phospholipids.

**Figure 6:**
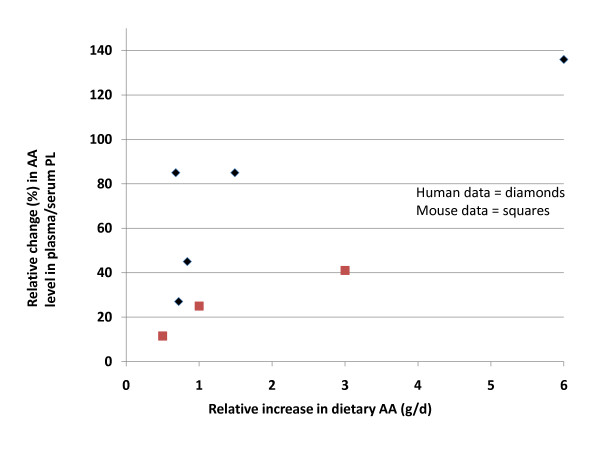
**Comparison of AA data in the mouse to similar data generated in human clinical trials; increasing levels of supplemented AA (% change based on energy) on changes in plasma/serum AA content**. The changes in AA levels of plasma phospholipids from this study (red squares) were plotted against archival data from human clinical trials (blue diamonds) (used with permission, see ref. 11). Abbreviations: AA, arachidonic acid; PL phospholipids.

When the mouse data for LA was plotted against similar data generated in human clinical trials based on % energy [11], the results were similar between species (Figure 5). When the mouse data for AA was plotted against similar data generated in human clinical trials based on % energy [11], the changes in AA levels in the mice resembled the human data at the two lowest doses but not at the highest dose (Figure 6).

## Discussion

Animal models are not intended to replace humans, but be a substitute that is often better controlled and better able to answer narrow research questions that could not be done, on a practical basis, with humans. A common challenge faced by nutrition researchers who are interested in interspecies comparisons is identifying an appropriate background diet and appropriate doses for supplemented nutrients. In order to make these choices, studies have to be performed that can demonstrate human equivalent responses to ensure translation between species. Without these fundamental studies, no guidelines can be formulated governing scientific justification for dosing when extrapolation to humans is desired. Currently, no guidelines exist for appropriate dosing of dietary PUFAs for experimental models (i.e., mice, rats) as they relate to humans and their intakes. As such, the overall objective of this research was to determine the extent to which supplementation of human equivalent doses of LA and AA changed tissue AA content within the context of a Western-type diet using a common experimental rodent model. These results were then compared to similar data generated from clinical trials with increasing and decreasing levels of dietary LA [11]. This is the first known study of its kind.

Interspecies relationships of body mass and mammalian physiology have been explored for over a century where various aspects of metabolism are proportional to an exponent of body weight *(W*^*n*^*)*, with "*n" *being between 0.67 and 0.75 [15-18]. Interspecies comparisons with regards to energetics were in part pioneered by Brody and Kleiber where they described the non-linear relationship between metabolic rate and body mass as it relates to allometric scaling (as reviewed by [19]). They described the concept that the relationship between metabolic rate (*MR*) and body weight could be linearized with the following equation: *MR = a(W *^*n*^*) *(where "*a*" is a proportionality constant, "*W" *is in Kg and *"n" *is an exponent between 0.70 and 0.75). More recently, Rucker and Storms (2002) elegantly described the pitfalls of using differences in body weight as a means of making interspecies extrapolations for micronutrients because of these non-linear relationships [20]. They addressed the appropriateness of several mathematical approaches to extrapolate nutrient intake between mice and humans and suggested food (energy) intake rather than body weight should be used to extrapolate nutrients for interspecies comparisons [20,21]. Interestingly, when this concept was applied to a variety of standardized semi-purified diets (i.e., AIN76A, AIN93G, AIN93M), extrapolations of the micro- and macronutrients (i.e., PUFA) better mimicked recommended intakes (i.e., the DRIs) when based on energy differentials as compared to body weight (Table 7 and Table 8). This provided the rationale, if not a scientific justification, for the background diet and doses used in this study.

**Table 7:** Allometric scaling of micronutrients in an AIN93G rodent diet based on differences in daily caloric consumption or body weight (kg) as compared to the daily recommended intakes for humans (DRI)

Nutrient	DRI	Kcal	BW
			
Thiamin	1.2 mg	15 mg	49 mg
Riboflavin	1.3 mg	2.7 mg	55 mg
B6	1.3 mg	3.0 mg	52 mg
Niacin	16 mg	15 mg	273 mg
Biotin	30 μg	101 μg	1820 μg
Folate	400 μg	1001 μg	18200 μg
Viatmin E	15 mg	38 mg	683 mg
Vitamin A	900 mg	605 mg	10,929 mg
Calcium	1000 mg	2601 mg	46,992 mg
Magnesium	400 mg	255 mg	4614 mg
Iron	8 mg	17 mg	315 mg
Manganese	2.3 mg	29 mg	533 mg
Selenium	55 μg	123 μg	2229 μg
Iodine	150 μg	105 μg	1893 μg
Zinc	11 mg	15 mg	266 mg

The data applies for a 25 g mouse consuming 15 Kcal/d and a 70 Kg human consuming 2000 Kcal/d. The conversion factor is 134 for calories (Kcal) and 2800 for body weight (BW).

**Table 8:** Allometric scaling of linoleic acid and alpha-linolenic acid in an AIN93G, AIN93M and AIN76A rodent diet based on differences in daily caloric consumption (Kcal) or body weight (BW) (in kg) as compared to the Adequate Intakes (AI) from the daily recommended intakes for humans (DRI)

Rodent Diet Fatty Acid	AI (DRI)	Kcal	BW
			
**AIN93G**^**a**^			
Linoleic acid	12-17 g	20 g	423 g
Alpha-linolenic acid	1.1-1.6 g	2.6 g	55 g
**AIN93M**^**b**^			
Linoleic acid	12-17 g	12 g	242 g
Alpha-linolenic acid	1.1-1.6 g	1.6 g	31 g
**AIN76A**^**c**^			
Linoleic acid	2-17 g	15.6 g	336 g
Alpha-linolenic acid	1.1-1.6 g	0 g	0 mg

The data applies for a 25 g mouse consuming 15 kcal/d and a 70 Kg human consuming 2000 Kcal/d.

^a^Based on a diet containing 7% (w/w) soybean oil that is 54% linoleic acid and 7% a-linolenic acid.

^b^Based on a diet containing 4% (w/w) soybean oil that is 54% linoleic acid and 7% a-linolenic acid.

^c^Based on a diet containing 5% (w/w) corn oil that is 60% linoleic acid.

With this in mind, we generated a "human equivalent" background diet where the macronutrient composition mimicked that of the human diet when based on energy (Table 9) and evaluated the impact of dietary LA (4%-14% of energy) and AA (0.08%-1.35% of energy) on changes in tissue AA levels in plasma and erythrocyte phospholipids. These amounts translate into human equivalent levels of 9-31 g/d and 0.18-2.7 g/d of LA and AA, respectively, and are within those ranges reported in the DRIs for humans and/or used in clinical trials [14,22]. Importantly, as opposed to rodent diets that selectively provide only one or two of the essential fatty acids (EFAs) (i.e., corn oil or soybean oil based diets), our background diet contained all the major n-6 and n-3 PUFAs found in the human diet (i.e., LA, ALA, AA and long chain n-3 PUFA). This is critical as all of these fatty acids are found in the Western diet and can have an impact on tissue AA levels. For this reason, there has been great interest placed upon n-6 PUFA metabolism, particularly when all EFAs are sufficiently provided in the diet at human equivalent levels.

**Table 9:** Allometric scaling of macronutrients in the US diet and the background diet of the mice used in this study based on caloric consumption

	US Diet	Rodent Diet
	%en; (g/d)	%en; (HED, g/d)
**Macronutrients**		
		
Protein	16%	16%
Carbohydrate	50%	50%
Lipids	34%	34%
SFA	13%	13%
MUFA	14%	14%
PUFA	7%	7%
		
**Polyunsaturated Fatty Acids**		
		
18:2 n-6	6.3%	6.3% (14 g)
18:3 n-3	0.6%	0.6% (1.3 g)
20:4 n-6	0.07%	0.07% (155 mg)
20:5 n-3	0.034%	0.034% (75 mg)
22:6 n-3	0.054%	0.054% (125 mg)

Abbreviations: HED, human equivalent dose.

The rodent model has been the superior target for investigation of specific fatty acids and downstream metabolites on tissue fatty acid composition since 1963 when Mohrhauer and Holman explored the metabolism of dietary EFAs [7]. In this classic and highly cited paper, rodents were initially fed a fat-free diet (i.e. with the exclusion of all EFAs) prior to supplementation with LA (ethyl linoleate) up to 5% from energy, where a 721% increase in liver AA composition was observed with the highest doses. Other studies recapitulated these earlier results when LA was provided to a background diet that lacked nearly all or completely all n-3 and n-6 PUFAs [9,10]. When LA was increased from nearly 0% of energy to 6-7% of energy in rodents, liver AA composition increased 173%-518% [6,8]. Increasing LA from 6% of energy to 27% of energy (or a human equivalent dose of 59 g/d) resulted in a 134% increase in tissue AA composition [5]. The addition of LA at supra-physiological doses (i.e., 17.3% of energy) from a background diet containing 7% LA, increased AA content by 375% when no other EFAs were provided [10]. However, when more moderate levels of LA were supplemented to a diet containing human equivalent levels (i.e., 6.6% to 13.2% of energy), AA content in liver phospholipids increased a modest 6% [8]. Notably, tissues have a requirement for unsaturated fatty acids for structural function and to help maintain membrane fluidity. When animals are fed a diet that exclusively contains a single PUFA (i.e., LA), its selective and robust conversion to a more highly unsaturated form is not surprising. These findings underscore the differential impact of dietary LA in rodent diets on changes in tissue AA content when the background diet is devoid of LA and/or other PUFAs, or providing LA at doses approaching pharmacological levels.

What is an appropriate background diet in rodents and what is an appropriate dose of LA that has translational ability to humans? This would be dependent upon the human literature; that is, what is the effect of LA on changes in tissue AA in individuals consuming a typical Western diet? The DRI (observed median intakes in the US population) for LA is 12 g/d and 17 g/d for women and men, respectively (approximately 6% of energy) [14]. In a recent review of the literature, decreasing LA content in the diet up to 90% or increasing the levels up to 550% was not associated with changes in AA content in the phospholipid pools of human plasma/serum (see Figure 5) or erythrocytes [11]. It is not unreasonable to think that with a background diet containing LA, ALA, AA, and long-chain n-3 PUFAs (i.e., EPA and DHA) at typical intakes, modifying LA levels may not influence tissue AA levels in these populations. Hence, in order to establish a human equitable response to dietary LA on tissue AA composition in the rodent model, it seems best accomplished when all EFAs are present in the diet, especially for results that are expected to translate proportionally to humans.

Increasing LA from 0% to 2% of energy replete tissue pools of n-6 PUFAs by increasing AA phospholipid concentrations [23]. Intake of LA above 2-3% of energy in humans is not reportedly accompanied by an increase in AA content in plasma or erythrocyte phospholipids [22,24-29], results consistent with our data. Poor conversion rates in humans would account for these results where the estimated fractional conversion of LA to AA in adults was between 0.3% and 0.6% [30]. In rodents, tracer kinetic analysis demonstrated greater efficiency of C20 fatty acids in conversion to downstream end-products relative to C18 precursors [31]. This would imply that feedback inhibition of Δ-6 desaturase, the rate limiting step in the conversion of LA to AA, may be responsible. Likewise, the present study reports no significant alteration in plasma/serum or erythrocyte AA phospholipids at the lowest supplemental dose of LA (4% of energy) or the highest supplemental dose (14% of energy). These results are supported by prior rodent dietary studies supplementing LA at similar levels (6.8% and 8.7% of energy) to a background diet already containing LA (4.5% of energy) [32,33]. Hence, supplementation of HEDs of LA to a background rodent diet consisting of all EFAs found in the human diet (including LA and AA), results in changes in tissue AA content that more accurately reflect those measured in humans consuming similar levels [22,24-26,29].

Additionally, our data demonstrate the observable inverse relationship of dietary AA and changes in tissue LA within the rodent model. When AA was supplemented to rodents consuming a Western-like diet, tissue AA content increased in a dose dependent manner, suggesting the lack of changes with LA supplementation was not due to saturation of AA in the phospholipid pools analyzed. Likewise, when dietary AA was provided to rodents (mice, hamsters) at 1.5-4% of energy, AA content in hepatic phospholipids increased 21-80%; [12,32-34]. Similar results were observed in intestines, macrophages, lung, heart, spleen, kidneys, testes and platelets [12,32,33]. These results are comparable to humans [11]; however, the response in rodents is more modest than that observed in humans supplemented with AA at the highest dose (see Figure 6) [11]. The estimated human intake for AA is <200 mg/day [4]. Our highest supplemented dose of 1.35% of energy (or a HED of 3 g/d) increased phospholipid AA levels in plasma/serum by 40%, while providing a dose of 0.75-1.5 g/d in humans increased tissue AA content by ~85% [35,36] with a maximum change of 136% at a dose of 6 g/d [37]. Rodents, compared to humans, have higher requirements for the more highly unsaturated fatty acid DHA in their tissue phospholipids [38]. This may preclude the need for higher levels of AA in tissues, accounting for the more modest effects observed in rodents following AA supplementation. Of importance, these changes in AA content were always at the expense of tissue LA, suggesting that dietary AA targets the same phospholipid pool occupied by LA [12,32,33]. This relationship between the changes in tissue levels of AA and LA following AA supplementation is supported by human clinical data [35]. The inverse is not always true. While some studies suggest an inverse relationship exists between tissue LA and AA levels when LA is supplemented in the diet [11,25,28], these studies are in the minority.

## Conclusions

Currently there are no guidelines providing assistance as to how to formulate a human equivalent diet for rodents to improve translation of data to humans. The overall intent of this research was to test a theoretical model for allometric scaling based on energy differences between species. We chose the relationship between dietary LA and its effects on tissue AA content as a testable target. We wanted to determine the extent to which supplementation with human equivalent doses of LA and AA changed tissue AA content within the context of a Western-type diet using a common experimental rodent model. We proposed that providing animals a background diet that mimicked the Western diet with regards to macro- and micronutrients and fatty acid profiles, and supplementing LA at human equivalent doses, we could observe a human equivalent response with regard to changes in AA levels in plasma/serum and erythrocyte phospholipids. Our results recapitulated those in humans and provide support for the concept that allometric scaling between species for dietary LA can be accomplished based on energy and metabolic differences. It is important to note that these results cannot be extrapolated to all tissues.

## List of abbreviations

AA: arachidonic acid; ALA: alpha-linolenic acid; DHA: docosahexaenoic acid; DRI: dietary reference intake; EFA: essential fatty acid; EPA: eicosapentaenoic acid; HED: human equivalent dose; LA: linoleic acid; PUFA: polyunsaturated fatty acid

## Competing interests

The authors declare that they have no competing interests.

## Authors' contributions

KW conducted the research and co-wrote the manuscript, and JW formulated and designed the research, co-wrote the manuscript and had final responsibility for all parts of the manuscript. All authors have read and approved the final manuscript.

## Funding

This research was funded in part by the Tennessee Agricultural Experiment Station, University of Tennessee, Knoxville, TN (JW).
